# The TB Surge intervention: an optimized approach to TB case-finding in Nigeria

**DOI:** 10.5588/pha.23.0039

**Published:** 2023-12-07

**Authors:** C. Ogoamaka, O. Bethrand, U. Lotanna, O. Chidubem, U. Sani, N. Nkiru, B. Mamman, E. Daniel, O. Chijioke, N. Oloruntobi, I. Austin, N. Debby, E. Rupert, O. Omosalewa, U. Emperor, A. Chukwuma

**Affiliations:** 1 KNCV Tuberculosis Foundation Nigeria, Abuja, Nigeria; 2 United States Agency for International Development, Abuja, Nigeria; 3 National Tuberculosis and Leprosy Control Programme, Abuja, Nigeria

**Keywords:** public health facilities, intensified TB case-finding, presumptive TB, TB diagnosis and treatment

## Abstract

**INTRODUCTION:**

TB remains one of the leading causes of death in Nigeria, and despite progress in treatment coverage, a 56% gap in national case notifications remains. This gap is attributable in part to underdiagnosis due to missed cases in health facilities. The TB Surge intervention presented an opportunity to address barriers to optimal case detection in public health facilities.

**METHODS:**

KNCV Nigeria implemented the TB Surge intervention under the USAID-funded TB-LON Project in 1,041 public facilities from June 2020 to September 2022. Trained ad-hoc staff screened hospital attendees, linked identified presumptive TB cases to diagnosis and confirmed TB cases to treatment. Data were reported using the Commcare application. Robust project monitoring was used to address gaps.

**RESULTS:**

Of a total of 12,195,874 hospital attendees screened for TB, 729,369 identified as presumptive TB were tested and 65,029 TB cases were diagnosed; 8% of the TB cases were children. Overall TB yield was 9%. Medical ward service delivery point had the highest TB yield of 21%. The number needed to test was 11 and the number needed to screen was 188.

**CONCLUSION:**

The TB Surge intervention was of strategic importance in addressing missed cases and barriers to prompt TB diagnosis in health facilities.

## Abstract (fr)

**INTRODUCTION:**

La TB reste l’une des principales causes de décès au Nigeria et, malgré les progrès réalisés en matière de couverture thérapeutique, il subsiste un écart de 56% dans la déclaration des cas à l’échelon national. Cet écart s’explique en partie par le sous-diagnostic dû aux cas manqués dans les centres de santé. L’intervention TB Surge a permis de lever les obstacles à la détection optimale des cas dans les centres de santé publique.

**MÉTHODES:**

KNCV Nigeria a mis en œuvre l’intervention TB Surge dans le cadre du projet TB-LON financé par l’USAID dans 1 041 centres publics de juin 2020 à septembre 2022. Le personnel ad hoc formé a dépisté les personnes présentes à l’hôpital, lié les cas de TB présomptifs identifiés au diagnostic et les cas de TB confirmés au traitement. Les données ont été communiquées à l’aide de l’application Commcare. Un suivi rigoureux du projet a permis de combler les lacunes.

**RÉSULTATS:**

Sur un total de 12 195 874 patients hospitalisés ayant fait l’objet d’un dépistage de la TB, 729 369 cas présumés de TB ont été testés et 65 029 cas de TB ont été diagnostiqués ; 8% des cas de TB étaient des enfants. Le rendement global de la TB était de 9%. Le point de prestation de services du service médical a enregistré le taux de TB le plus élevé (21%). Le nombre de tests nécessaires était de 11 et le nombre de dépistages nécessaires de 188.

**CONCLUSION:**

L’intervention TB Surge a été d’une importance stratégique pour traiter les cas non détectés et les entraves à un diagnostic rapide de la TB dans les centres de santé.

TB was ranked as the world’s leading cause of death from an infectious agent until the COVID-19 pandemic.^1^ In 2021, 10.6 million people were estimated to have fallen ill with TB, with 6.8 million diagnoses and 1.6 million deaths.^2^ The number of deaths has risen by approximately 300,000 compared to 2021,^3^ despite the global target of achieving a 90% reduction in mortality by 2030,^4^ as outlined in the UN Sustainable Development Goals (SDGs), and the WHO's End TB strategy, which aims to achieve a 75% reduction in deaths by 2025.^5^

Africa accounted for 23% of individuals who contracted TB, and Nigeria, as the highest-burden country on the continent, ranked sixth worldwide, contributing 4.4% to the global estimate of incident cases.^2^,^6^ Despite improvement in 2021, the treatment coverage rate in Nigeria remains below 50% and there is a 6.3% gap between incident and reported new cases globally.^2^,^7^ These gaps were attributed to underreporting of diagnosed cases and underdiagnosis, both due to poor access to healthcare.

Missed TB cases contribute significantly to the continuing high prevalence and incidence of TB, particularly in low- and middle-income countries (LMICs) like Nigeria.^8^,^9^ Evidence indicates that the majority of TB cases that go undiagnosed are individuals who actively seek healthcare services.^10^ Persons with TB-related symptoms seeking care are regularly missed.^11^,^12^ Facility-based intensified case-finding (ICF) remains a highly potent and efficient means of finding missed TB cases.

Due to the high clinic attendance in public facilities, TB Surge sits at the core of achieving the USAID’s Local Organizing Network (LON 1&2) project implemented by KNCV Nigeria, main objective of which is finding and notifying missing TB cases. TB Surge is operational in 1,041 health facilities, at the primary, secondary and tertiary levels. TB Surge is an optimized ICF strategy in public facilities through a scale-up of systematic TB screening of all hospital attendees and modifications to the conventional process aimed at addressing existing barriers to optimal case detection of missed TB cases in health facilities.

This paper presents the intervention’s innovative case-finding practices in TB-LON 1 & 2-supported public facilities.

## METHOD

### Intervention

TB Surge is an ongoing intervention implemented in 14 Nigerian states comprising Akwa Ibom, Anambra, Bauchi, Benue, Cross River, Delta, Imo, Kaduna, Kano, Katsina, Nasarawa, Plateau, Rivers, and Taraba, cutting across the eastern, northern, and southern parts of Nigeria. TB Surge was anchored on the hub-and-spoke model and guided by a provider-initiated systematic case-finding framework (Figure).

**FIGURE:**
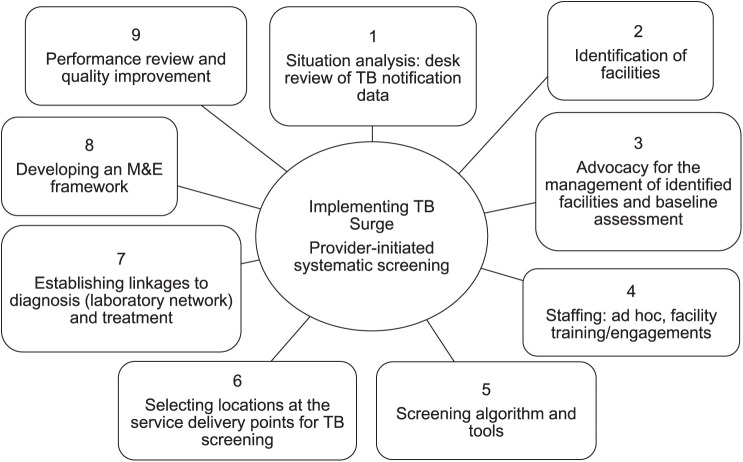
Framework for implementing TB Surge. M&E = monitoring and evaluation.

Implementation commenced with a situational analysis of TB-related service delivery and extensive baseline assessment in the implementing states. This was followed by site assessments, resource mapping, site identifications, and engagements. Facility identification was guided by high clinic attendance. Starting with 840 facilities in 2020, the number of engaged sites was gradually scaled up to 1,041 in 2022. Facility engagement was preceded by high-level advocacy to site management, with the active collaboration of key stakeholders at the local, state, and national TB programs. Stakeholders’ buy-in facilitated the seamless implementation of the intervention.

Ad hoc staff – TB screeners, data clerks, and linkage coordinators – were recruited from a pool of experienced healthcare workers in the local communities to facilitate local acceptance. Having a dedicated staff for TB screening reduced the workload on hospital staff. Facility-level training was conducted to build and strengthen their capacities in TB service delivery. To build sustainability and to provide TB services outside of work hours and weekends, facility health workers were also trained. Comprehensive refresher training courses were conducted at intervals to reinforce learnings via KNCV Nigeria’s competency-based training (CBT) platform. All currently engaged ad hoc staff were required to obtain this certification to continue to offer TB services.

Keying into the hub-and-spoke model, the spokes feed into the laboratory and DOTS services of the hubs clustered within a locality. The spokes comprise primary-level facilities with high clinic attendance whose health workers were trained to screen and identify presumptive TB cases and link these to diagnosis in the hub facilities. The hubs comprise the secondary and tertiary hospitals, often with diagnostic and treatment facilities. Active TB screening also occurs at the hub sites; a linkage coordinator works within each cluster to ensure the linkage of presumptive TB and diagnosed TB cases to treatment, as well as proper documentation. This model not only ensures timely retrieval of results and treatment initiation, but a patient-centered approach to TB care as well.

At the least, a TB screener is domiciled in each of the hospital service delivery points (SDPs) and clinics to screen all consenting persons, both patients and non-patients, who arrive at the hospital daily. More than one TB screener is engaged in SDPs with high attendance. High-risks group clinics such as those on antiretroviral therapy, nutrition and diabetics are prioritized. TB screening is done using the WHO 4-symptom screen (cough, fever, night sweats, and weight loss). In children, additional symptoms of neck and back swelling, and failure to thrive are recorded. The screening tools, register, and Commcare app are designed to aid health workers in symptom grading and presumptive identification. The Commcare app is also used to electronically provide case-by-case patient longitudinal tracking from screening to treatment commencement. Commcare works on mobile devices and can be used in low network coverage settings as it allows for offline data capture .

Patients who are unable to provide sputum are provided with free X-ray coupons and transportation support, including transportation fares, for clinical evaluation at project-engaged X-ray sites. In children, non-invasive stool-based expert testing is used. The sample referral network and health model for sample transportation and timely retrieval of results were adopted. In case of delays, the linkage coordinators or project logistic teams are mobilized for sample transportation to mitigate sample wastage.

The project monitoring and evaluation framework robustly tracks and reports data across the TB cascade, from clients screened to presumptive TB identified, and patients diagnosed and enrolled for treatment. Through the project summary template, data are collated weekly and disaggregated by relevant data elements and dimensions such as organization units (facility, local government administration [LGA], state), age, sex, type of TB, and diagnostic method. Weekly performance is assessed during a project-wide review meeting, where implementation outlook is presented using dashboards and other visuals. This informs the programmatic decisions and strategies going into the new week. High-frequency project monitoring and data reporting are undertaken to identify gaps, followed by root cause analysis, designing interventions and strategies to address the identified gaps, and periodic program learning to share best practices. Monthly quality improvement and data review meetings provide opportunities for quality checks on reported data and processes by the state project team, discussion of implementation challenges, review of strategies, and reinforcement of learning for optimal implementation. This is further complemented by technical assistance visits and quarterly data quality assessment exercises to the states by the central project team.

### Data collection

Data clerks, domiciled at the facilities, collect and report case-based data daily through the Commcare app. Updates on evaluation and treatment status are also sent through the platform. Facility-based aggregated data are reported weekly through project summary templates. These data are validated at different levels of the project – LGA, state and national. Data is collected across the TB cascade.

### Data analysis

A descriptive analysis was conducted to assess the performance of the TB Surge intervention between June 2020 to September 2022. Cascade efficiencies were presented on a year-by-year project cycle basis to present performance trends. The first-year project cycle covered only 4 months (June–September). Independent variables such as age and sex, were also extracted and stratified. Outcome variables were presented in the form of counts and proportions to provide a detailed performance outlook.

### Ethical consideration

The study was determined to be a non-research program evaluation. As it required no direct contact with human subjects (no interview or sample collection), and only de-identified pooled program data that formed part of standard of care were used, informed consent was not required.

## RESULTS

A total of 1,041 facilities were engaged for the TB Surge intervention between June 2020 and September 2022, representing a 24% scale-up from the start of the project. During this period, 12,195,874 hospital attendees were screened (Table 1), yielding 803,916 (7%) presumptive TB cases. Of the persons presumed to have TB, 729,369 (91%) were evaluated and 65,029 were diagnosed (TB yield: 9%); 61,989 (95%) were initiated on treatment and notified to the national TB program. The number needed to test (NNT) was 11, and the number needed to screen (NNS) was 188.

**TABLE 1:** TB Surge efficiency cascade

Period	Person screened *n*	Presumed to have TB *n* (% presumptive yield)	Presumptive evaluated for TB *n* (% evaluation rate)	TB patients *n* (% TB yield)	Started on TB TX *n* (% enrolment rate)	NNT	NNS
June–September 2020 (Year 1)	1,068,445	65,711 (6)	55,588 (85)	6,189 (11)	5,169 (84)	9	173
October 2020–September 2021 (Year 2)	5,441,684	379,542 (7)	341,628 (90)	28,481 (8)	27,338 (96)	12	191
October 2021–September 2022 (Year 3)	5,685,745	358,663 (6)	332,153 (93)	30,359 (9)	29,482 (97)	11	187
Total	12,195,874	803,916 (7)	729,369 (91)	65,029 (9)	61,989 (95)	11	188

NNT = number needed to test; NNS = number needed to screen.

Assessment of yearly contributions showed a steady increase across cascades in absolute numbers as the year progressed. The number of presumptive TB cases identified and tested increased significantly from 2020 to 2021, but dipped slightly in 2022. In terms of efficiency, TB yield was highest in 2020 (11%) and lowest in 2021 (8%), whereas the enrolment rate was highest (97%) in 2022 and least (84%) in 2020. NNT and NNS were lowest in 2020 and highest in 2021.

The results of the intervention are given by sex and age in Table 2. More women were screened for TB than men, but more men were diagnosed with TB across the 2 years and 4-month period. Specifically, 39% of persons screened were men, while 59% of diagnosed cases were men. Individuals aged 25–34 years represented the highest number of individuals screened and diagnosed for TB than other age brackets. This was followed by the 35–44 year age group. Children represented 23% of persons screened. Childhood TB (0–14 years) stood at 8%, 9%, and 7% for the years 2020, 2021, and 2022, respectively. Although only 41% of the children screened were between 5 and 14 years, more TB cases (62%) were diagnosed in this age bracket.

**TABLE 2.** TB Surge yearly cascade disaggregated by sex and age

Indicator	Sex		Age, years								Total
	Male	Female	0–4	5–14	15–24	25–34	35–44	45–54	55–64	≥65	
June–September 2020 (Year 1)											
Clients screened for TB	372,310	696,135	116,056	70,048	160,924	288,225	203,123	106,210	69,469	54,390	1,068,445
Clients presumed to have TB	27,368	38,343	3,554	4,426	10,298	15,307	12,645	8,293	5,803	5,385	65,711
Presumptive cases evaluated for TB	23,023	32,565	2,849	3,577	8,941	13,413	10,808	6,835	4,754	4,411	55,588
Clients diagnosed with all forms of TB	3,672	2,517	177	314	1,063	1,406	1,232	924	602	471	6,189
All forms of TB patients started on treatment	3,218	1,951	138	251	940	1,166	1,022	775	484	393	5,169
October 2020–September 2021 (Year 2)											
Clients screened for TB	2,099,388	3,342,296	779,783	503,578	858,987	1,151,216	851,972	568,324	411,008	316,816	5,441,684
Clients presumed to have TB	171,812	207,730	28,419	36,351	56,644	73,827	65,595	49,481	37,044	32,181	379,542
Presumptive cases evaluated for TB	154,539	187,089	24,506	31,916	51,350	67,346	59,747	44,438	33,444	28,881	341,628
Clients diagnosed with all forms of TB	16,729	11,752	974	1,474	4,014	6,699	6,213	4,209	2,661	2,237	28,481
All forms of TB patients started on treatment	16,128	11,210	888	1,401	3,861	6,441	5,989	4,060	2,550	2,148	27,338
October 2021–September 2022 (Year 3)											
Clients screened for TB	2,305,804	3,379,941	770,992	567,617	902,063	1,110,730	869,150	631,317	467,295	366,581	5,685,745
Clients presumed to have TB	163,462	195,201	25,949	34,875	53,747	68,805	61,428	48,242	35,343	30,274	358,663
Presumptive cases evaluated for TB	151,960	180,193	23,049	31,568	49,478	63,986	57,672	45,288	32,930	28,182	332,153
Clients diagnosed with all forms of TB	18,224	12,135	808	1,364	4,244	7,127	6,660	4,781	2,987	2,388	30,359
All forms of TB patients started on treatment	17,699	11,783	782	1,323	4,158	6,932	6,420	4,645	2,906	2,316	29,482

TB Surge was implemented across the hospital SDPs. The analysis of SDPs (Table 3) indicated that in the second and third years in the project cycle, TB yield among presumptive TB cases tested was highest in the medical ward at 17.3% and 21%, respectively. The medical ward also had the highest efficiency, with an NNT of 5 and NNS of 68 between 2021 and 2022; this was followed closely by the General Out Patient Department (GOPD) with NNT of 7 and NNS of 131. There was no TB screening in the diabetes clinic at the start of the project.

**TABLE 3:** TB yield across the service delivery points

Service delivery points	June–September 2020 (Year 1)			October 2020–September 2021 (Year 2)			October 2021–September 2022 (Year 3)		
	TB yield %	NNT	NNS	TB yield %	NNT	NNS	TB yield %	NNT	NNS
Accident and emergency	14.1	7	101	10.3	10	174	8.8	11	156
Antenatal care	5.6	18	577	4.1	25	782	4.9	20	792
Antiretroviral therapy clinic	9.1	11	164	8.5	12	185	10.1	10	153
Child welfare	4.2	24	688	9.1	11	346	4.0	25	281
Diabetes clinic	0.0	0	0	10.8	9	276	13.2	8	158
General outpatient department	14.0	7	132	13.4	7	135	14.2	7	131
Immunization	4.5	22	1454	8.9	11	970	6.7	15	1456
Medical ward	7.0	14	292	17.3	6	84	21.1	5	68
Medical outpatient department	9.9	10	274	10.2	10	262	10.1	10	250
Others	14.3	7	138	10.9	9	205	10.8	9	260
Pediatric outpatient department	8.6	12	252	9.1	11	274	10.4	10	233

NNT = number needed to test; NNS = number needed to screen.

## DISCUSSION

The findings from the review of the TB Surge implementation show it contributed to reaching persons with TB who passed through the public health facilities. Through the engagement of well-trained facility and ad hoc staff (TB screeners, data clerks, and linkage coordinators), TB Surge was able to address the common barriers to case detection in facilities such as healthcare workers’ low TB suspicion index,^13^ inadequate use of diagnostic algorithms, and poor case-finding coordination,^14^ suboptimal TB screening, low testing capacity, poor grasp of the screening protocols, and low diagnostic capacities,^8^,^15^,^16^ high patient load and untrained staff,^17^ inadequate staff strength and misdiagnosis.^18^

TB Surge showed the effectiveness of prioritizing public facilities with high clinic attendance in the provision of TB services to reach a critical mass of the population. According to a study in Kampala, Uganda, this is a cost-effective TB strategy for TB control, increasing the proportion of presumptive TB cases by 6% and newly diagnosed TB cases by 30% at a low cost.^19^ The high TB yield aligns with findings in other literature,^20^,^21^ and can be attributed to its high access to patients.

The first period/fiscal year (June to September 2020) only had an implementation period of 4 months in 840 facilities, contributing to the low screening numbers. With the full-year implementation and strategic expansion of supported facilities and provision of services in all the SDPs in the other fiscal years, more people were screened. The enhancement of service quality and the enforcement of best practices significantly contributed to greater achievements across the cascade in 2022 compared to previous years. The increase in the evaluation rate was the result of adopting strategies that emphasized the enhancement of sample quality, the reduction of turnaround time in sample handling, testing, and result retrieval. These strategies included implementing the sample referral network and healthcare worker model, as well as making ad-hoc arrangements in exceptional situations. New diagnostic tools – TB LAMP (Eiken Chemical; Tokyo, Japan) and TrueNat® (Molbio Diagnostics, Verna, India) were also deployed to improve diagnostic network and ensure prompt diagnosis. The treatment enrolment rate increased steadily between 2020 and 2022, in part, due to timely tracking of diagnosed patients and linkage to care and proper documentation of patients’ biodata at first contact.

More males were diagnosed with TB, although more females were reached through screening; this is in line with other study findings.^22^–^24^ This underscores the health-related challenges faced by men and their limited utilization of healthcare services.^25^ It may also be the result of a higher tendency among men to engage in greater health-risk behavior such as alcohol, substance, and tobacco abuse, than women.^26^ Factors influencing the risk of TB such as comorbidity predominance, alcohol abuse, history of incarceration, and smoking, were more frequent in males than females.^27^ Another study reported the incidence rate of pulmonary TB to be higher among men.^28^ More females visited healthcare facilities than males, as evidenced by their higher number of screenings. This contradicts claims of low TB diagnosis among women, suggesting that barriers to healthcare facility access for economically disadvantaged women may not be as significant as previously thought.^23^

The breakdown by age groups revealed that among children, although those aged 0–4 years underwent more screening, TB was actually more prevalent in the 5–14-years age group, aligning with findings from another study.^29^ Additionally, TB was more common among individuals in the productive age range (those who are sexually active) of 25–34 years and 35–44 years, as also observed in a separate study.^23^,^30^ Both age groups represent the highest number of people who were screened.

Across the period under review, the medical ward and the GOPD presented a higher TB yield and efficiencies, with the least NNT and NNS compared to other SDPs. This shows the high yield potential at these SDPs, probably because of the very high patient throughput. Prioritizing 100% screening in these clinics will continue to boost TB yield for the intervention.

Our study had some limitations. The data collection within the TB program is still manually based, and some omissions could occur during data collection and transcription into the CommCare App; however, monthly data quality assessments were conducted to ensure data accuracy. A limited number of identified presumptive TB cases were not evaluated due to inadequate diagnostics tools; this was improved by instituting sample referral mechanisms.

## CONCLUSION

The TB Surge intervention presented a vital opportunity to find and treat the missing TB cases passing through public health facilities that would have otherwise remained undetected. With TB Surge, a modified ICF intervention, optimal screening was provided to all hospital attendees, both patients and caregivers. Barriers to optimal diagnosis were adequately addressed using best practices. The strategic approaches adopted in the implementation of this intervention are highly recommended, as these ensure that all the critical components of TB service delivery – case finding, diagnosis, treatment, and reporting – are optimized.
